# Supplementary material for: Real-Time Observation of Clickable Cyanotoxin Synthesis in Bloom-Forming Cyanobacteria Microcystis aeruginosa and Planktothrix agardhii
Source: Toxins (Basel). 2024 Dec 5;16(12):526. doi: 10.3390/toxins16120526 (PMC11679549; doi:10.3390/toxins16120526)
Supplement: Supplementary file 1 [file toxins-16-00526-s001.zip › toxins-3281476-supplementary.pdf]

Supplementary Material

Article

Real-Time Observation of Clickable Cyanotoxin Synthesis in Bloom-Forming Cyanobacteria *Microcystis aeruginosa* and *Planktothrix agardhii*

Rainer Kurmayer * and Rubén Morón Asensio

Research Department for Limnology, University of Innsbruck, Mondseestrasse 9, 5310 Mondsee, Austria; ruben.moron-asensio@uibk.ac.at
* Correspondence: rainer.kurmayer@uibk.ac.at

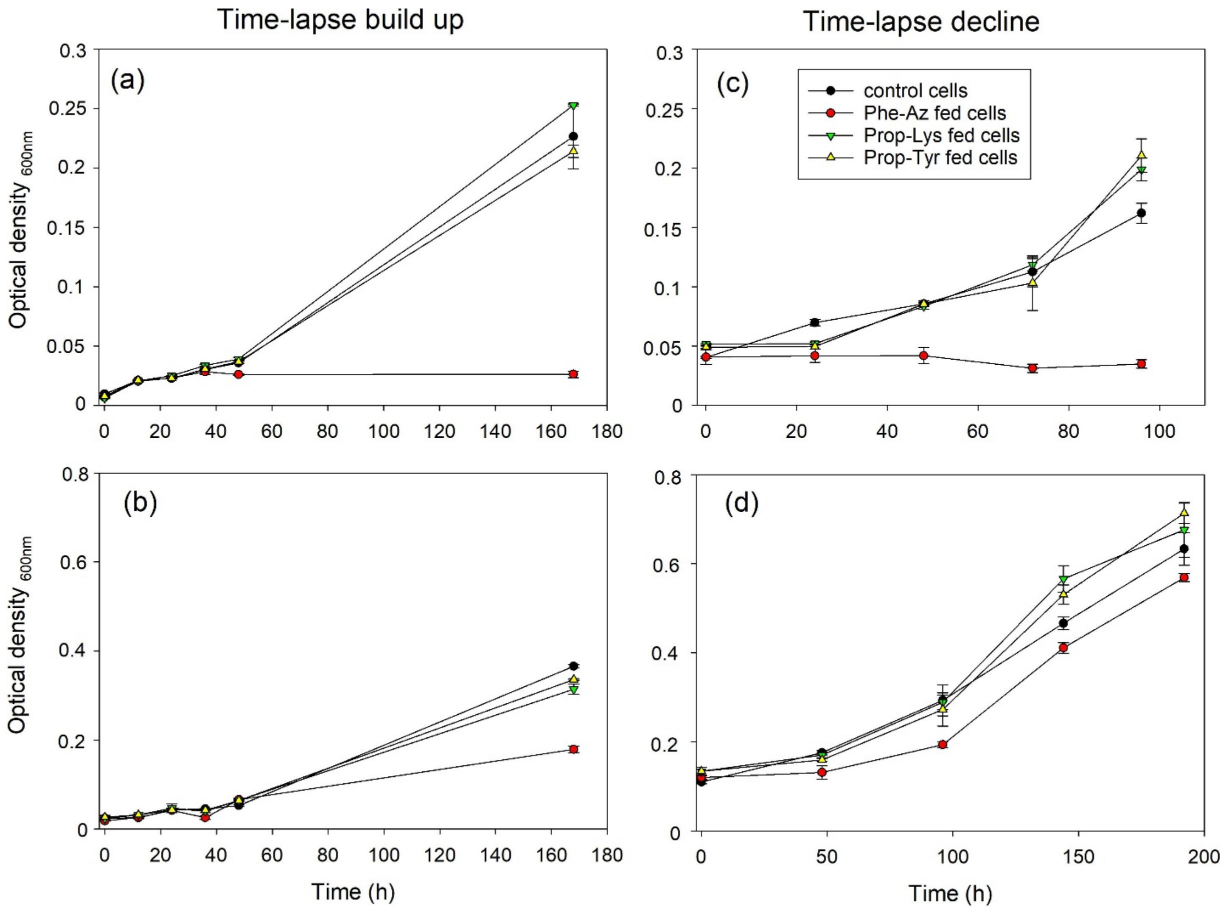

Supplementary Figure S1: Mean (± SE) optical density of strain cultures of (a, c) *M. aeruginosa* strain Hofbauer and (b, d) *P. agardhii* strain no371/1 during time-lapse experiments using pulsed feeding of non-natural amino acids (non-AAs) under maximum growth rate conditions in order to observe the build up (a, b) or decline (c, d) of azide- or alkyne-modified MC or AP peptides.

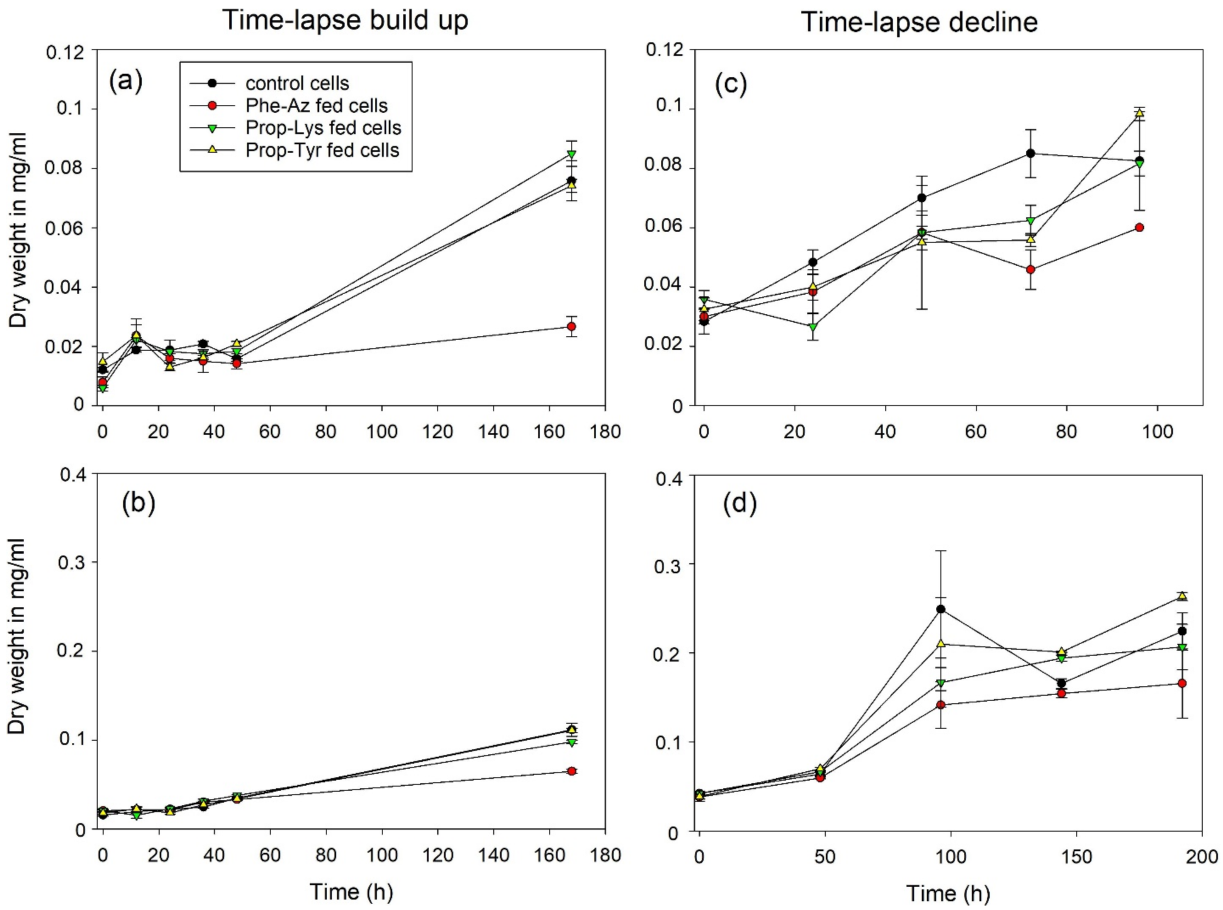

Supplementary Figure S2: Mean (± SE) dry weight (mg/ml) of strain cultures of (a, c) *M. aeruginosa* Hofbauer and (b, d) *P. agardhii* no371/1 during time-lapse experiments using pulsed feeding of non-natural amino acids (non-AAs) under maximum growth rate conditions in order to observe the build up (a, b) or decline (c, d) of azide- or alkyne-modified MC or AP peptides.

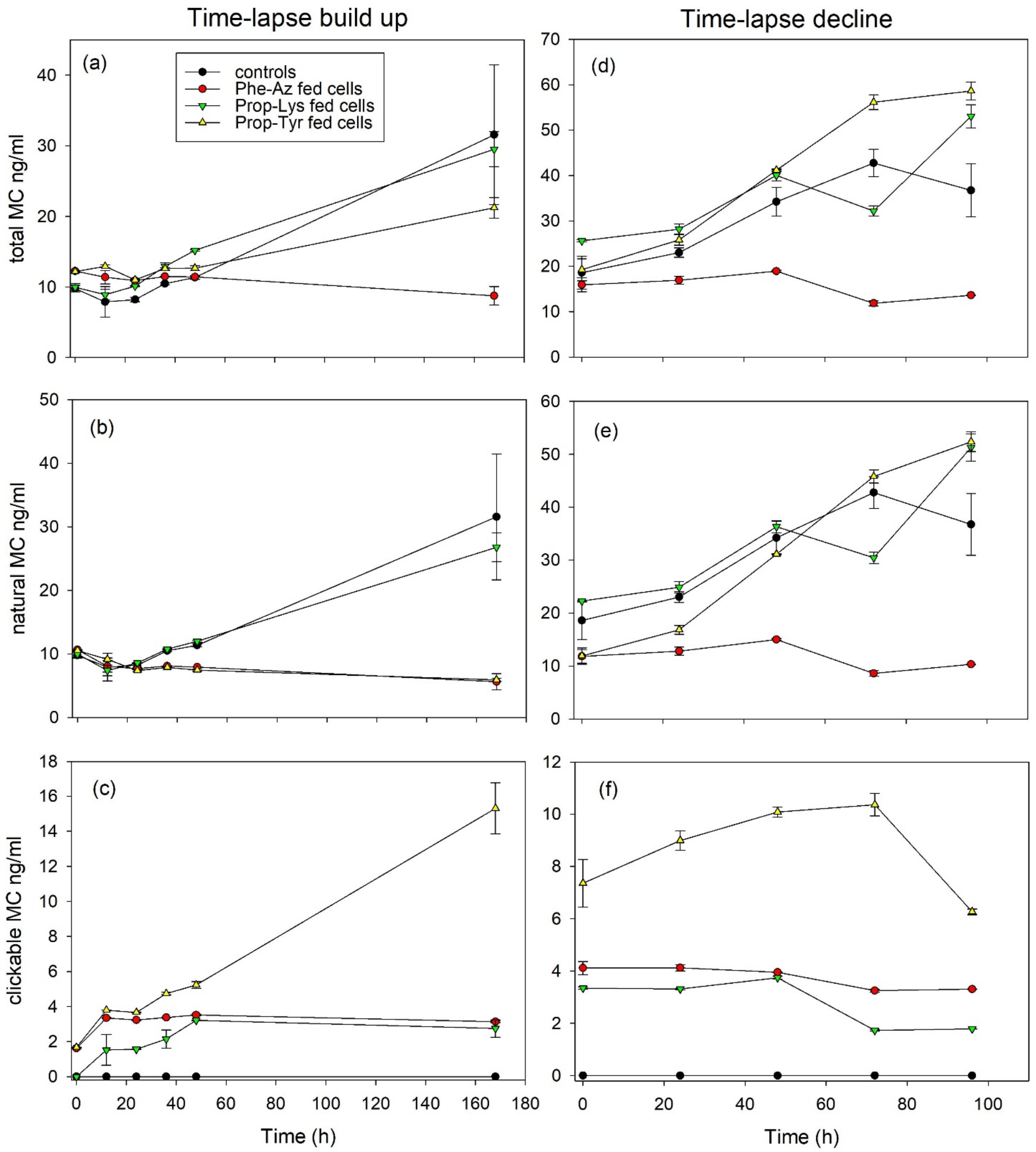

Supplementary Figure S3: Mean (± SE) cellular concentration of (a, d) total, (b, e) natural and (c, f) clickable MC in ng of MC-LR equiv. /ml (composed of four MC structural variants: DAsp-MC-YR, MC-YR, DAsp-MC-LR, MC-LR) during time-lapse experiments using pulsed feeding of non-natural amino acids (non-AAs) in order to observe the increase (c) or decline (f) of azide- or alkyne-modified MC in *M. aeruginosa* strain Hofbauer.

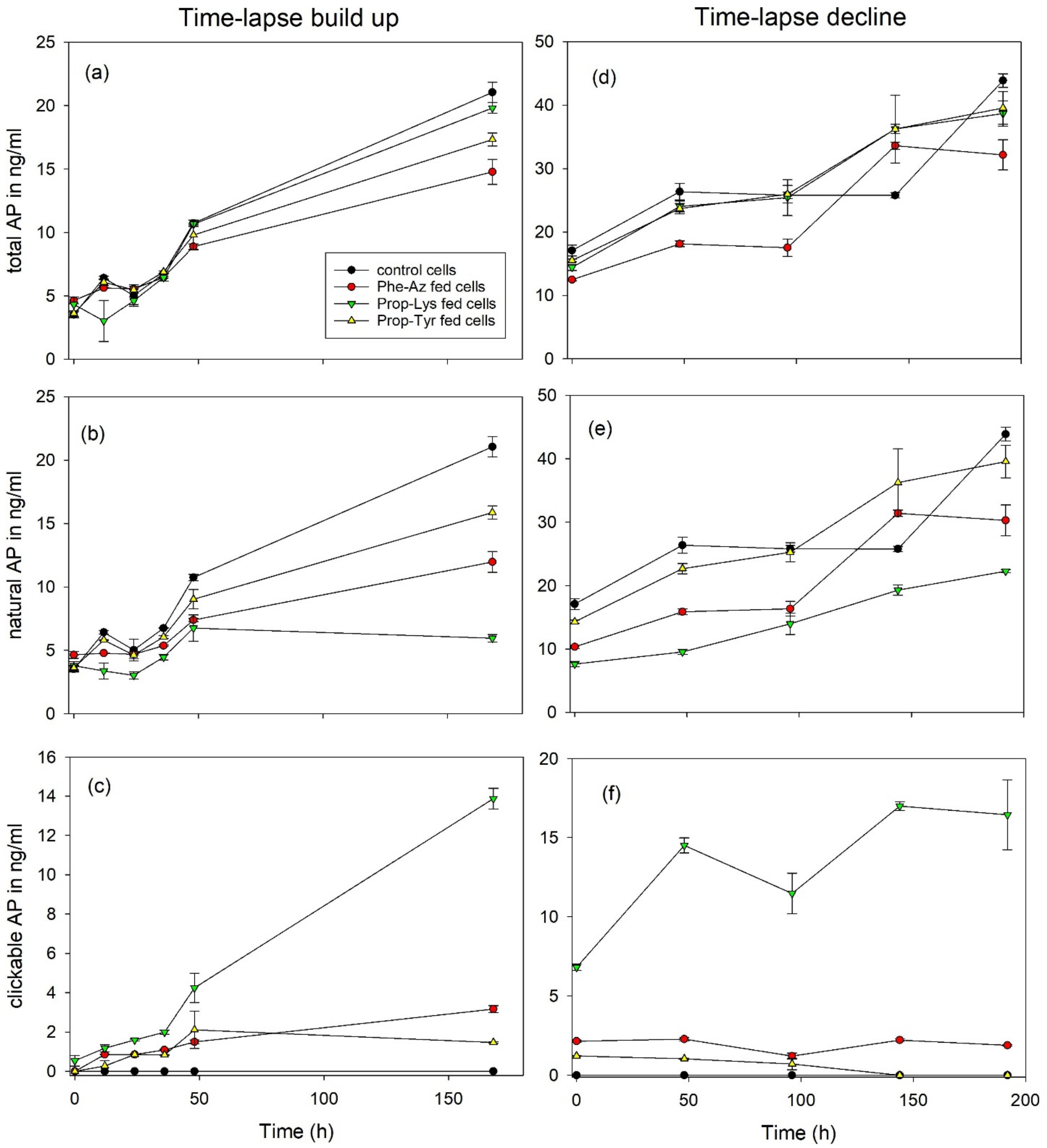

Supplementary Figure S4: Mean (± SE) cellular concentration of (a, d) total, (b, e) natural and (c, f) clickable AP in ng of AP-B equivalents /ml (composed of four AP structural variants: AP (unknown), AP-C, AP-B, AP-A) during time-lapse experiments using pulsed feeding of non-natural amino acids (non-AAs) in order to observe the increase (c) or decline (f) of azide- or alkyne-modified AP in *P. agardhii* strain no371/1.

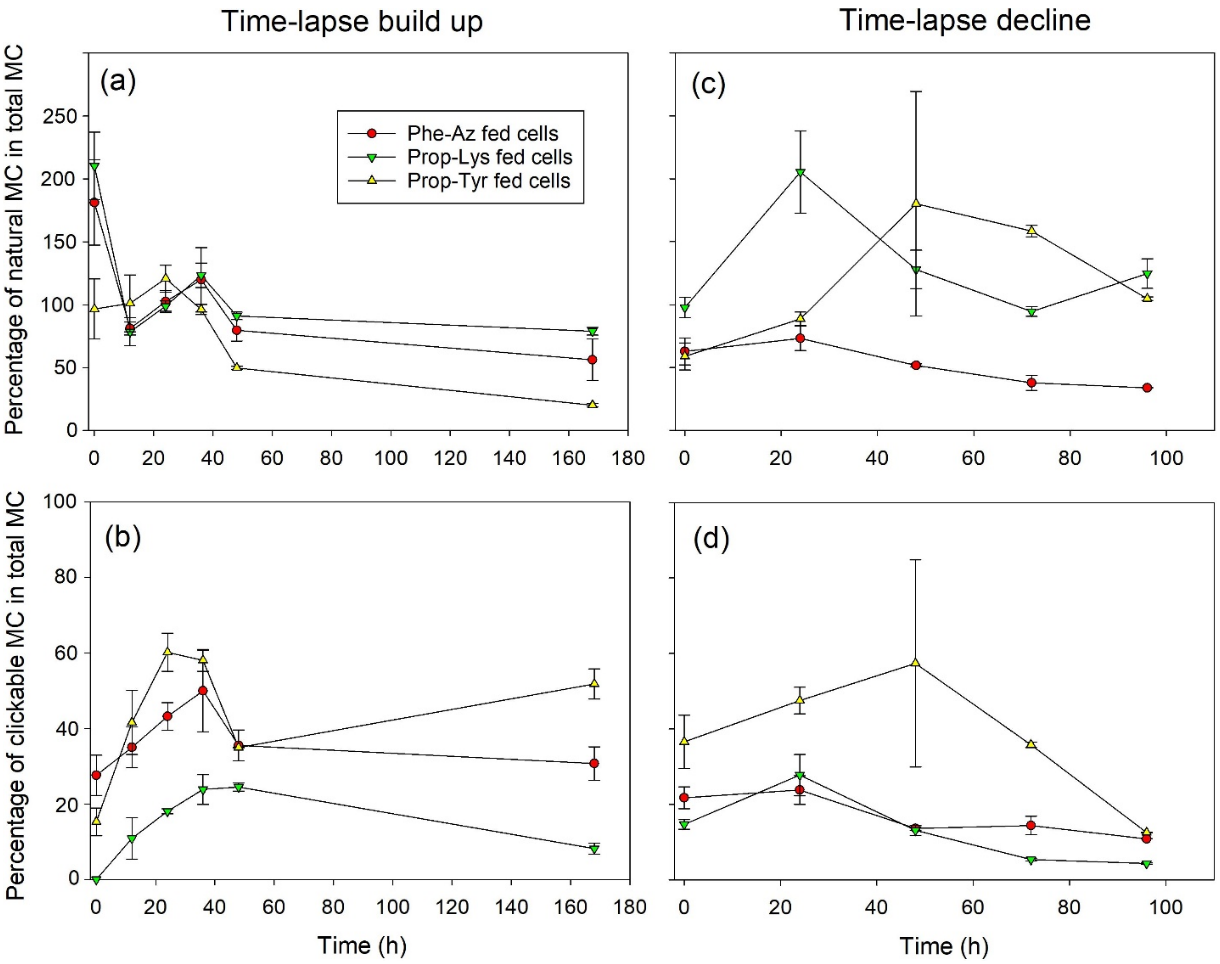

Supplementary Figure S5: Mean (± SE) cellular content of natural and clickable MC in percentage of control in *M. aeruginosa* strain Hofbauer (ng MC-LR equiv./ mg DW, composed of four MC structural variants: DAsp-MC-YR, MC-YR, DAsp-MC-LR, MC-LR) during time-lapse experiments using pulsed feeding of non-natural amino acids (non-AAs) in order to observe the build up (a, b) or decline (c, d) of azide- or alkyne-modified MC. (Control = cells grown and processed under identical conditions but without non-AA substrate).

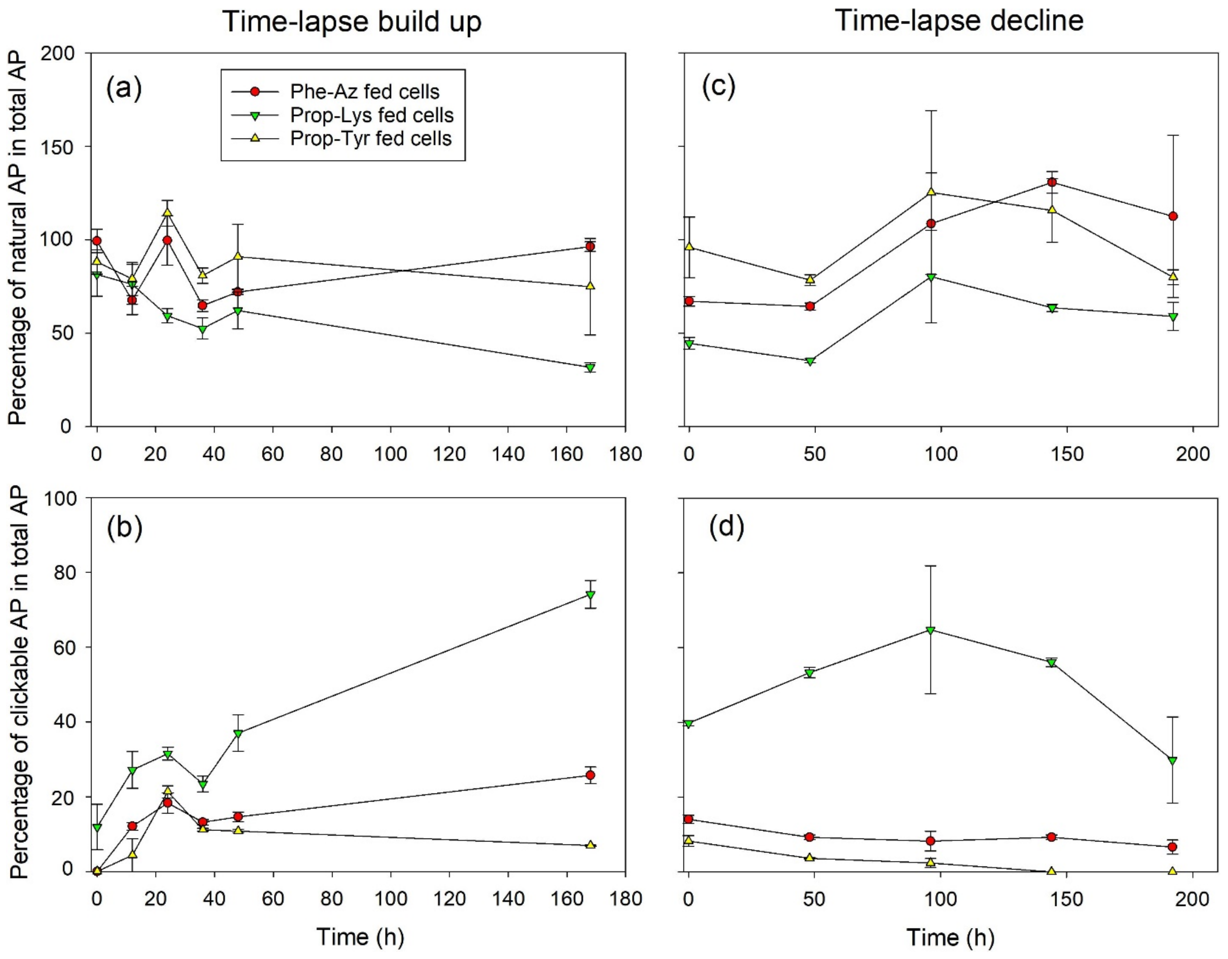

Supplementary Figure S6: Mean (± SE) cellular proportion of natural and clickable AP in total AP in percentage of control in *P. agardhii* strain no371/1 (ng AP-B equiv./ mg DW, composed of four AP structural variants: AP unknown, AP-C, AP-B, AP-A) during time-lapse experiments using pulsed feeding of non-natural amino acids (non-AAs) in order to observe the build up (a, b) or decline (c, d) of azide- or alkyne-modified AP. (Control = cells grown and processed under identical conditions but without non-AA substrate).

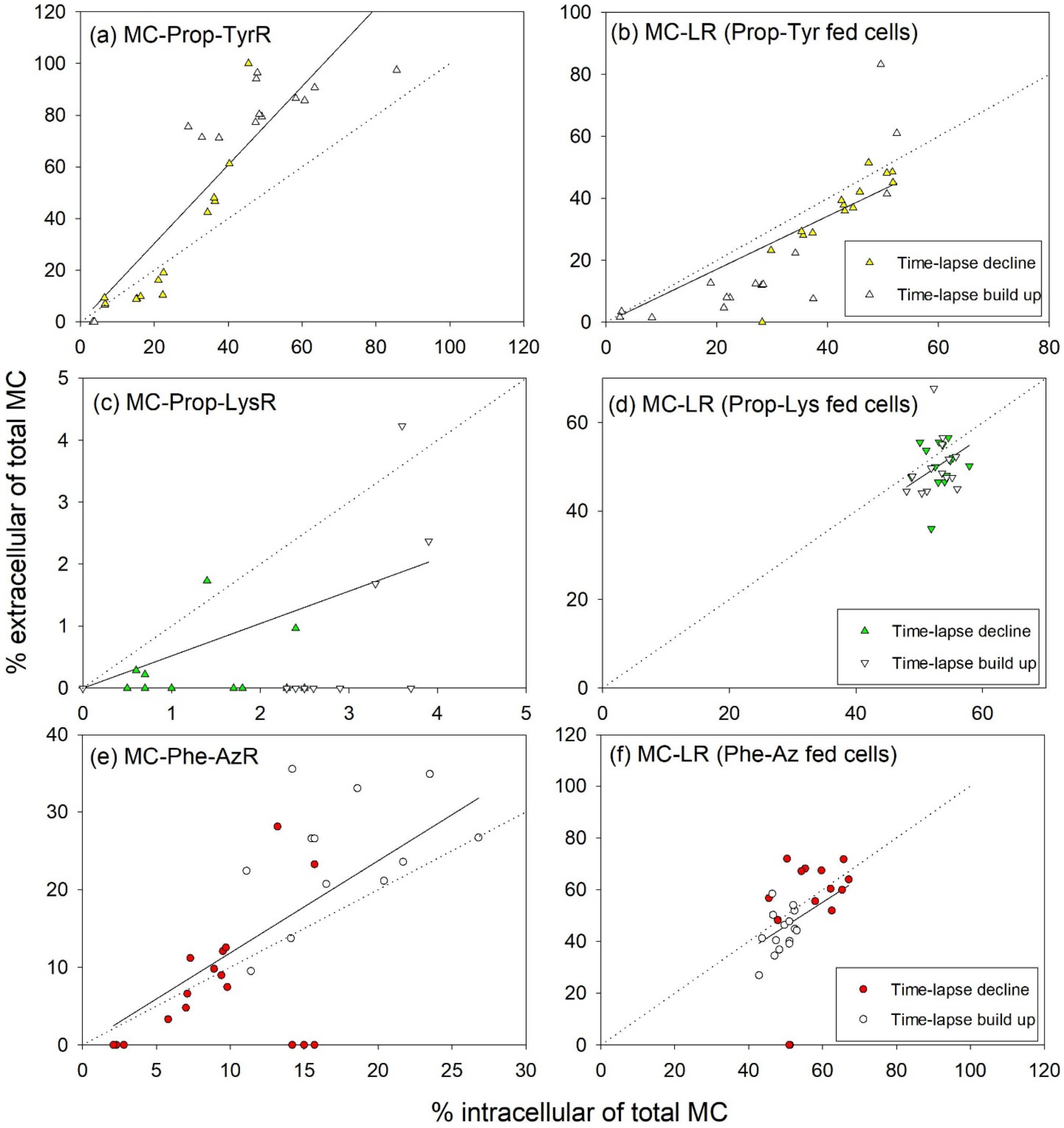

Supplementary Figure S7: Percentage of clickable and natural MC peptides in dissolved vs. intracellular fraction in *M. aeruginosa* strain Hofbauer during time-lapse experiments using pulsed feeding of non-natural amino acids (non-AAs) in order to observe the build up (open symbols) or decline (closed symbols) of azide- or alkyne-modified MC. Details of regression curves: (a) MC-Prop-TyrR ($R^2 = 0.94$), $y = 1.5186x$; (b) MC-LR (Prop-Tyr fed cells), ($R^2 = 0.87$), $y = 0.856x$; (c) MC-Prop-LysR ($R^2 = 0.39$), $y = 0.523x$; (d) MC-LR (Prop-Lys fed cells), ($R^2 = 0.99$), $y = 0.9467x$; (e) MC-Phe-AzR ($R^2 = 0.8$), $y = 1.1864x$; (f) MC-LR (Phe-Az fed cells), ($R^2 = 0.91$), $y = 0.9185x$, where y was percentage of extracellular MC in total extracellular MC and x was percentage of intracellular MC in total intracellular MC. The dotted line represents the 1:1 reference line.

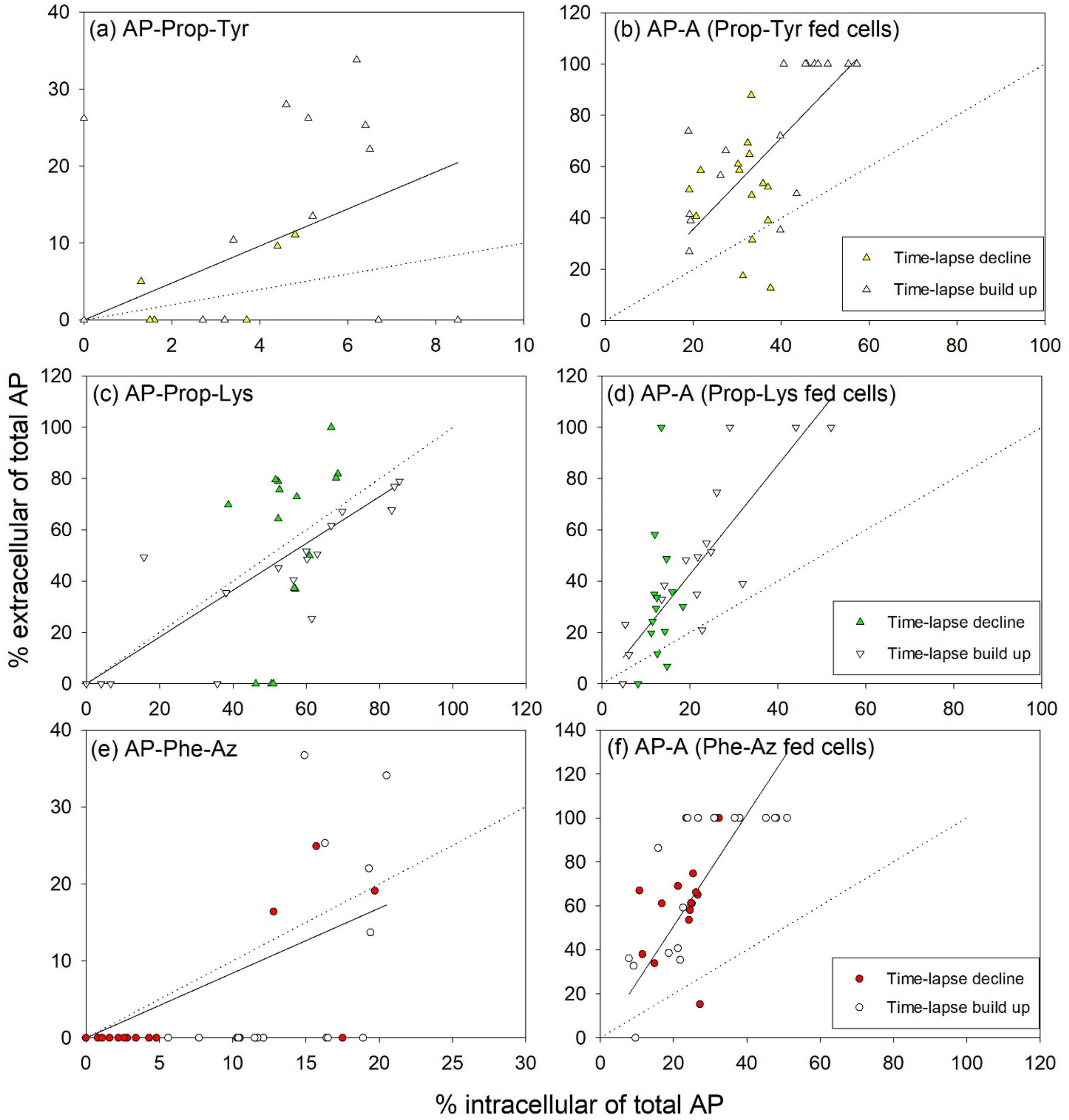

Supplementary Figure S8: Percentage of clickable and natural AP peptides in dissolved vs. intracellular fraction in *P. agardhii* strain no371/1 during time-lapse experiments using pulsed feeding of non-natural amino acids (non-AAs) in order to observe the build up (open symbols) or decline (closed symbols) of azide- or alkyne-modified AP. Details of regression curves: (a) AP-Prop-Tyr ($R^2 = 0.48$), $y = 2.4073x$; (b) AP-A (Prop-Tyr fed cells), ($R^2 = 0.9$), $y = 1.78x$; (c) AP-Prop-Lys ($R^2 = 0.83$), $y = 0.913x$; (d) AP-A (Prop-Lys fed cells), ($R^2 = 0.85$), $y = 2.133x$; (e) AP-Phe-Az ($R^2 = 0.51$), $y = 0.842x$; (f) AP-A (Phe-Az fed cells), ($R^2 = 0.91$), $y = 2.542x$, where y was percentage of extracellular AP in total extracellular AP and x was percentage of intracellular AP in total intracellular AP. The dotted line represents the 1:1 reference line.

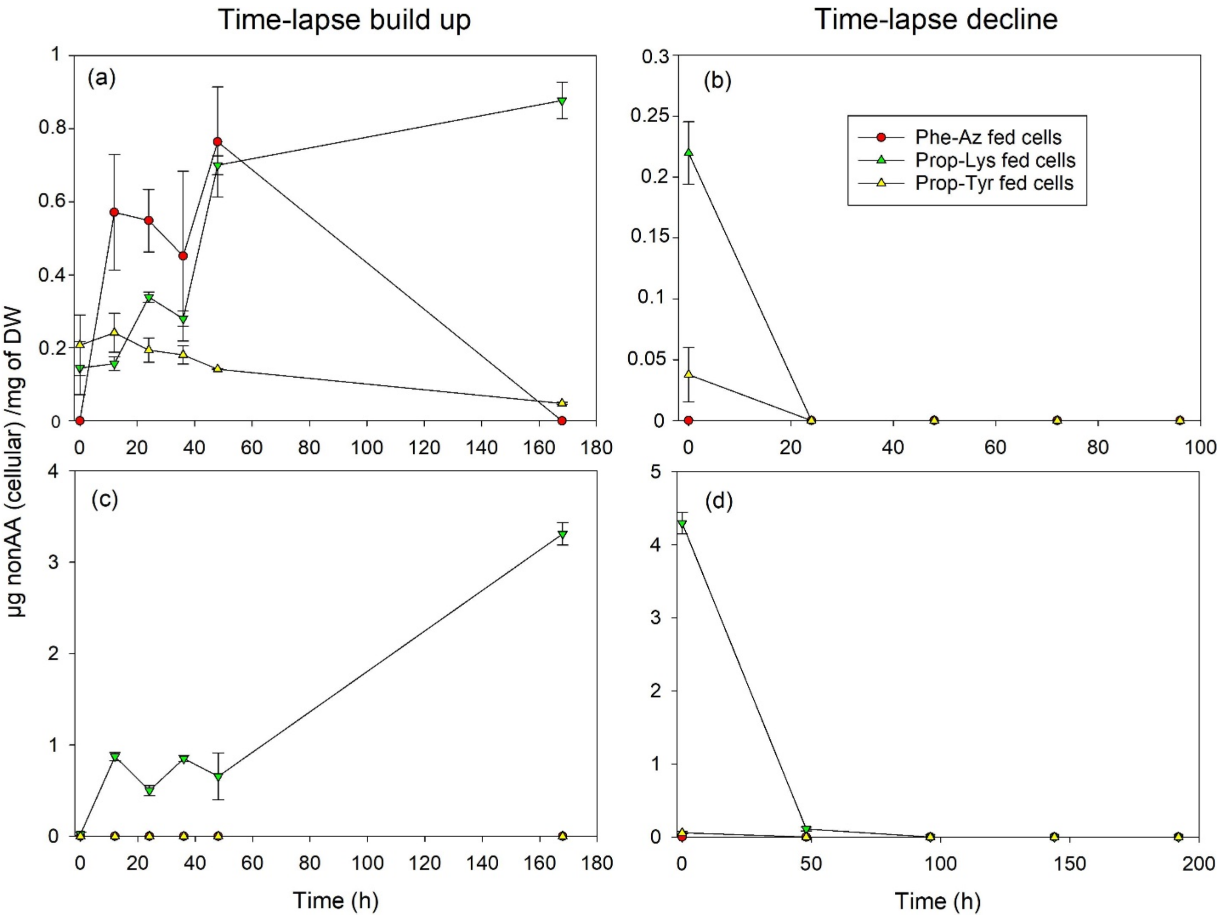

Supplementary Figure S9: Mean ± SE cellular content of non-AAs (Phe-Az, Prop-Lys, Prop-Tyr) eluting at 3.4 min (Prop-Lys), 5.2 min (Prop-Tyr), 5.4 min (Phe-Az) in µg of non-AA per mg of DW in peptide extract during time-lapse experiments using pulsed feeding of non-AAs in order to observe the build up (a, c) or decline (b, d) of azide- or alkyne-modified MC/AP peptide in *M. aeruginosa* (a, b) and *P. agardhii* (c, d).

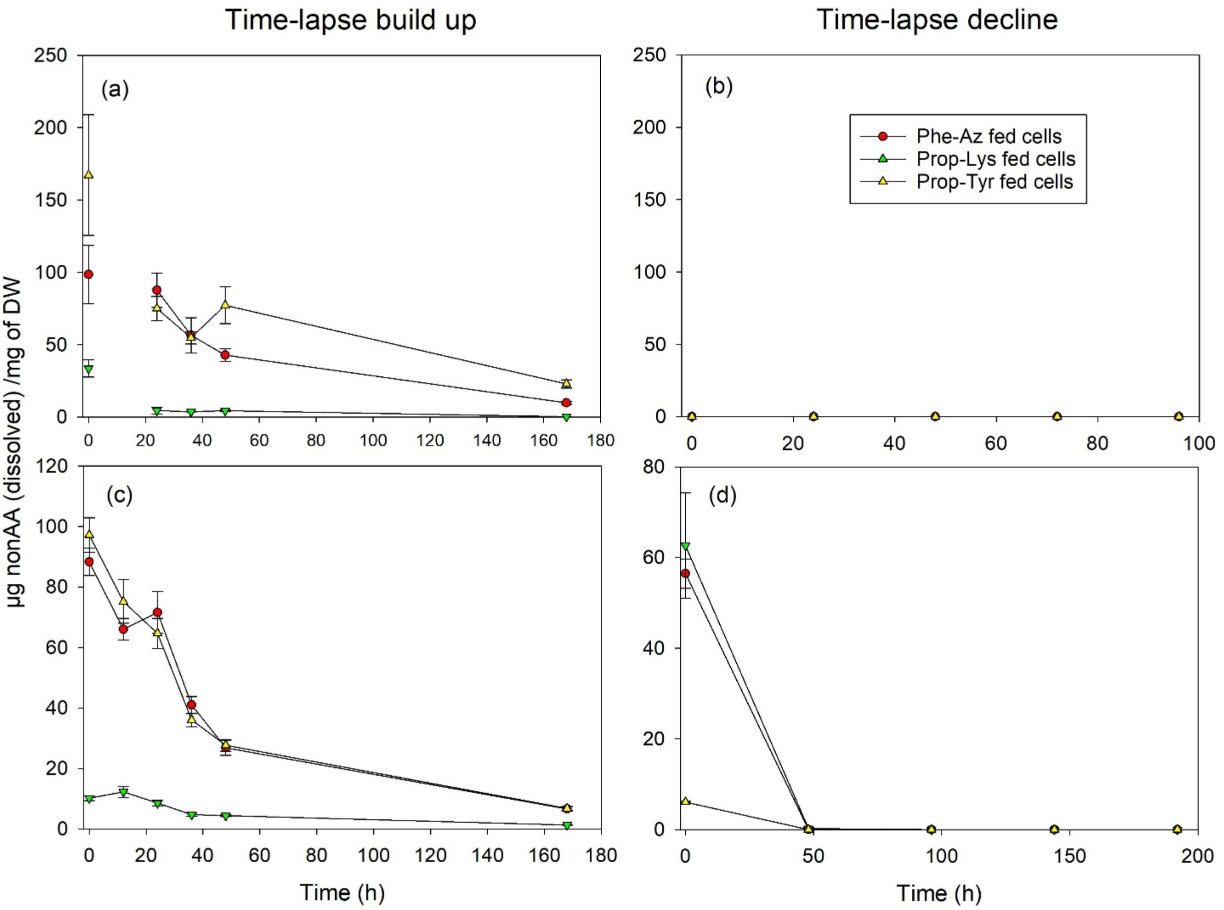

Supplementary Figure S10: Mean ± SE dissolved quantity of non-AAs (Phe-Az, Prop-Lys, Prop-Tyr) eluting at 3.4 min (Prop-Lys), 5.2 min (Prop-Tyr), 5.4 min (Phe-Az) in µg of non-AA per mg of DW during time-lapse experiments using pulsed feeding of non-AAs in order to observe the build up (a, c) or decline (b, d) of azide- or alkyne-modified MC/AP peptide in *M. aeruginosa* (a, b) and *P. agardhii* (c, d). Explanation for missing data in (a): No SPE measurements were performed at T1 (12 h).

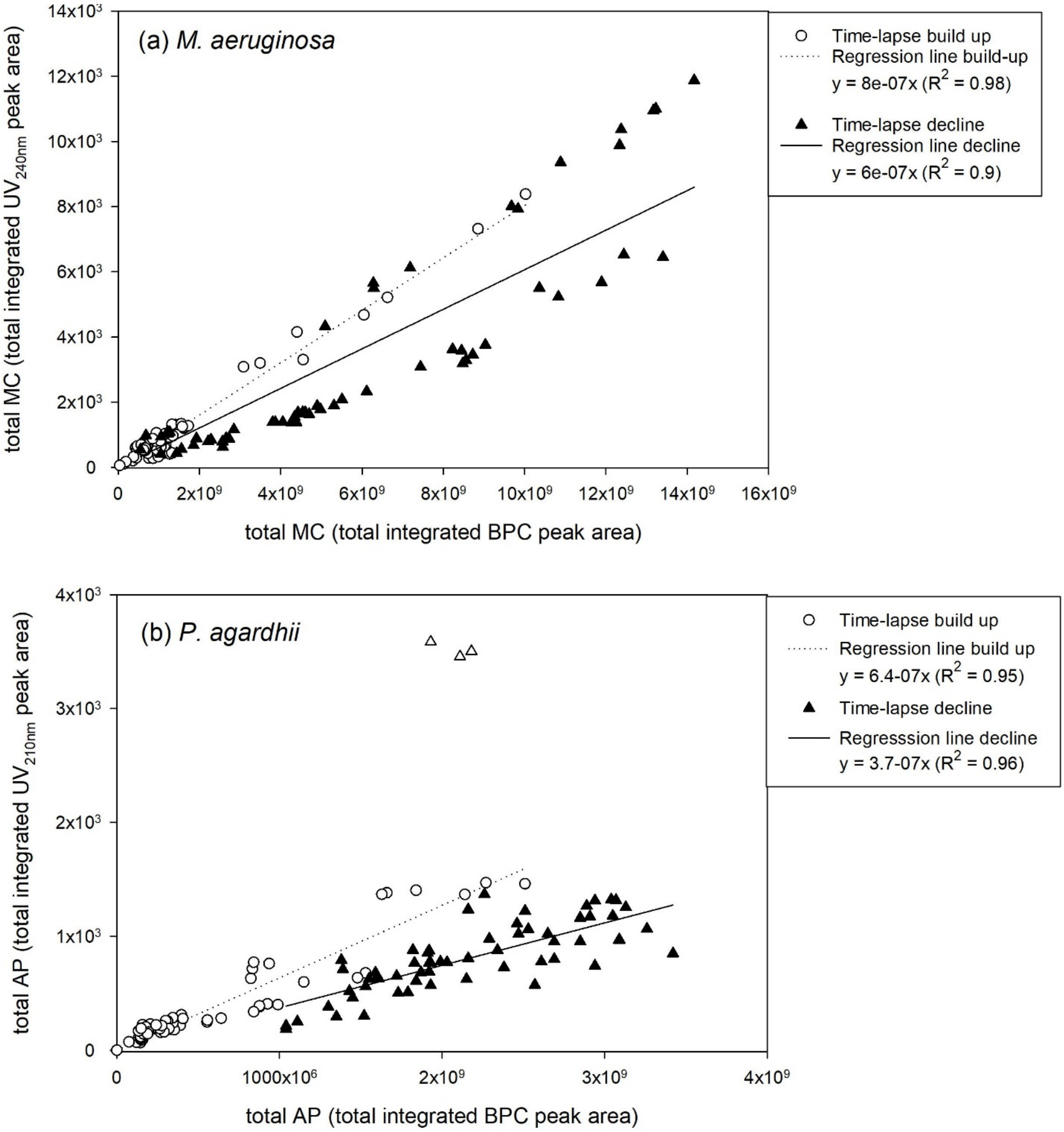

Supplementary Figure S11: Comparison of total MC (a) or (b) AP peptide quantification using either MS peak area (base peak chromatograms, BPC or extracted ion chromatograms, EIC) or UV peak area (at 240 nm for MC or 210 nm for AP) during all time-lapse experiments. In (b) three outliers (white triangles) were omitted from calculating the regression line for build up experiments.
